# Improving Carer Recognition and Understanding of Constipation for People with Intellectual Disability: Quality Assurance of an Online Learning Resource

**DOI:** 10.1192/bjo.2025.10319

**Published:** 2025-06-20

**Authors:** Perlie Pui Lum Tse, Rhiannon Lewis, Catherine Walton

**Affiliations:** 1 Cardiff University, Cardiff, United Kingdom; 2 Swansea Bay University Health Board, Swansea, United Kingdom.; 3 Swansea Bay University Health Board, Cardiff, United Kingdom

## Abstract

**Aims:** People with Intellectual Disability (PwID) have a reduced life expectancy in comparison to the general population, and constipation has been identified as a contributing factor to mortality by the Learning Disability Mortality Review. As part of a broader Quality Improvement project seeking out ways to reduce the rate of constipation for PwID it was recognised that robust and long-term education of carers was lacking.

An online learning resource was created ‘Constipation in PwID for Social Carers’ to support those caring for PwID to recognise and appropriately signpost constipation-related issues.

The learning resource was created by healthcare professionals, and it was therefore deemed necessary to undertake Quality Assurance of the module to ensure it was appropriate in both content and tone for support workers working in the social care sector. The final module was developed therefore with the input of those it was aiming to teach.

**Methods:** Focus groups and feedback forms collected information regarding carer’s role, experience working with PwID, understanding and relevance of learning outcomes, overall quality, and suggested improvements of the learning resource. This was undertaken in focus groups, or on a one-to-one basis. Feedback forms were completed by 12 individual participants. Focus groups involved 20 participants total including community nurses, supported living managers, support workers, social workers, and occupational therapists.

**Results:** Overall, the quality of the draft learning resource was rated ‘excellent’ and the general feedback was that it was appropriately pitched for carers. Aspects of the draft that helped with understanding content involved the use of scenario based interactive questions and visual aids. The information which was considered most useful included the Bristol Stool Chart, red light signs of constipation, and statistics on the prevalence of constipation in PwID.

Suggested improvements for language were consistent terminology for PwID, and avoiding medical jargon to keep advice applicable in different settings. Participants asked for clear communication of the responsibility of support workers to escalate information to supported living management, GP, and NHS 111.

**Conclusion:** Undertaking a robust Quality Assurance exercise for this online learning resource has ensured that language and terminology is appropriate for the target audience. Participants requested a clear message about how to escalate concerns. The next step will be to publish the resource online and evaluate its effectiveness in improving knowledge of constipation for carers of PwID.

